# 2024 UPDATE: the Brazilian Diabetes Society position on the management of metabolic dysfunction-associated steatotic liver disease (MASLD) in people with prediabetes or type 2 diabetes

**DOI:** 10.1186/s13098-024-01259-2

**Published:** 2024-01-19

**Authors:** Amélio F. Godoy-Matos, Cynthia Melissa Valério, Wellington S. Silva Júnior, João Marcello de Araujo-Neto, Marcello Casaccia Bertoluci

**Affiliations:** 1 Sociedade Brasileira de Diabetes (SBD), São Paulo, Brazil; 2 Instituto Estadual de Diabetes e Endocrinologia do Rio de Janeiro (IEDE), Rio de Janeiro, RJ Brazil; 3 Endocrinology Discipline, Department of Medicine I, Faculty of Medicine, Center of Biological Sciences, Universidade Federal do Maranhão (UFMA), Praça Gonçalves Dias, 21, Centro, São Luís, MA CEP 65020-240 Brazil; 4 Universidade Federal do Rio de Janeiro (UFRJ), Rio de Janeiro, RJ Brazil; 5 Universidade Federal do Rio Grande Do Sul (UFRGS), Porto Alegre, RS Brazil

**Keywords:** Metabolic dysfunction-associated steatotic liver disease, Fatty liver, Type 2 diabetes, Management, Guidelines, Pioglitazone, GLP-1RA

## Abstract

**Background:**

Metabolic dysfunction-associated steatotic liver disease (MASLD) is the most common liver disease affecting 30% of the world’s population and is often associated with metabolic disorders such as metabolic syndrome, type 2 diabetes (T2D), and cardiovascular disease. This review is an update of the Brazilian Diabetes Society (*Sociedade Brasileira de Diabetes [SBD]*) evidence-based guideline for the management of MASLD in clinical practice.

**Methods:**

The methodology was published previously and was defined by the internal institutional steering committee. The SBD Metabolic Syndrome and Prediabetes Department drafted the manuscript, selecting key clinical questions for a narrative review using MEDLINE via PubMed with the MeSH terms [diabetes] and [fatty liver]. The best available evidence was reviewed, including randomized clinical trials (RCTs), meta-analyses, and high-quality observational studies related to MASLD.

**Results and conclusions:**

The SBD Metabolic Syndrome and Prediabetes Department formulated 9 recommendations for the management of MASLD in people with prediabetes or T2D. Screening for the risk of advanced fibrosis associated with MASLD is recommended in all adults with prediabetes or T2D. Lifestyle modification (LSM) focusing on a reduction in body weight of at least 5% is recommended as the first choice for these patients. In situations where LSMs are insufficient to achieve weight loss, the use of anti-obesity medications is recommended for those with a body mass index (BMI) ≥ 27 kg/m^2^. Pioglitazone and glucagon-like peptide-1 receptor agonists (GLP-1RA) monotherapy are the first-line pharmacological treatments for steatohepatitis in people with T2D, and sodium–glucose cotransporter-2 (SGLT2) inhibitors may be considered in this context. The combination of these agents may be considered in the treatment of steatohepatitis and/or fibrosis, and bariatric surgery should be considered in patients with a BMI ≥ 35 kg/m^2^, in which the combination of LSM and pharmacotherapy has not been shown to be effective in improving MASLD.

## Introduction

**Fig. 1:**
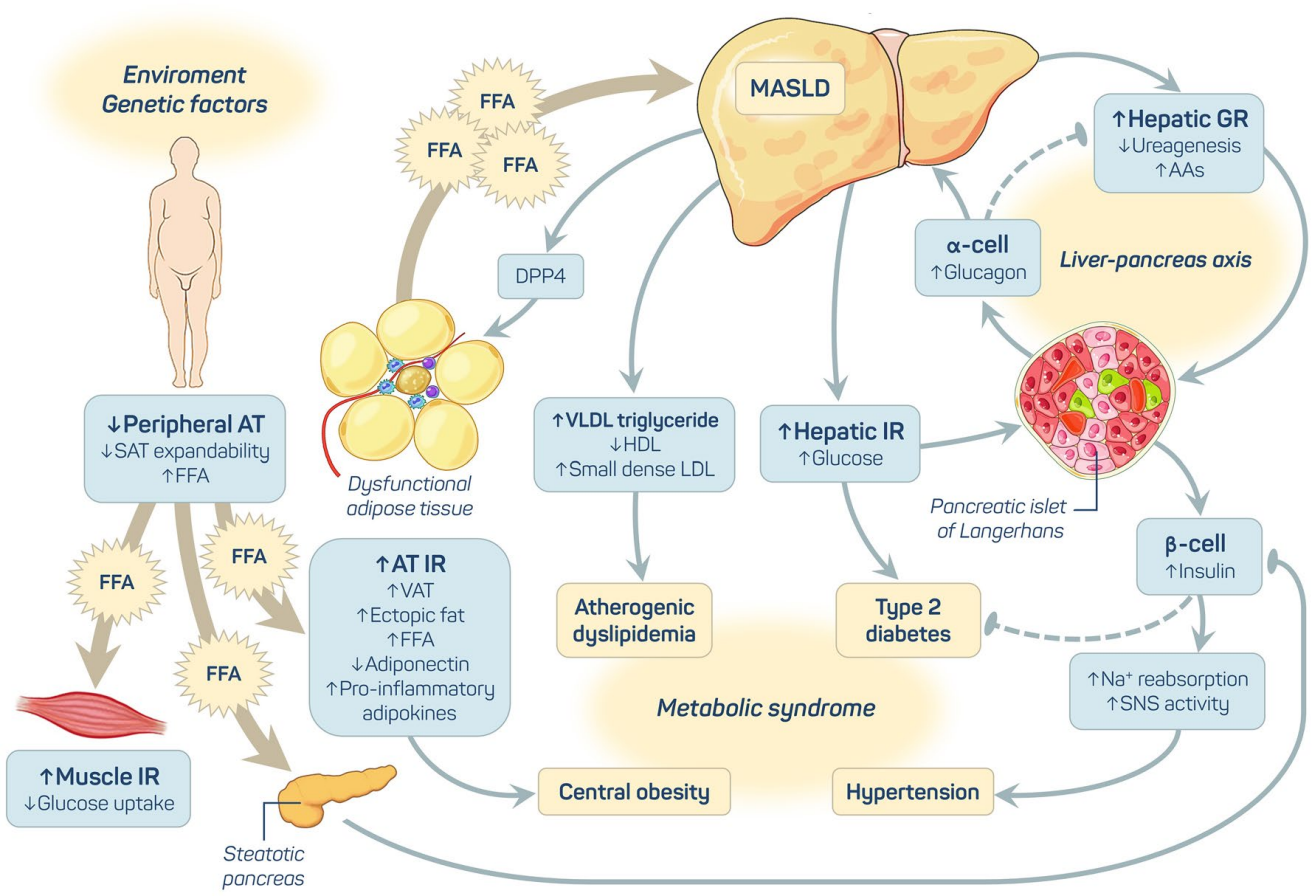
Pathophysiology of metabolic dysfunction-associated steatotic liver disease (MASLD). Environmental factors affect the expression of genes, inducing weight gain. When the capacity to expand subcutaneous adipose tissue (AT) is reached, increased free fatty acid (FFA) deposition occurs in visceral and ectopic sites. One ectopic site is the muscle, where increased FFA deposition promotes insulin resistance (IR), inhibiting insulin-mediated glucose uptake. On the other hand, AT insulin resistance facilitates lipolysis and increases the flux of FFAs to the liver, inducing hepatic IR and enhancing glucose production, de novo hepatic lipogenesis, VLDL release and atherogenic dyslipidemia. FFAs spill over into the pancreas, facilitating β-cell dysfunction through lipotoxicity, hyperglycemia and diabetes (the twin cycle hypothesis). Increased liver fat also promotes hepatic glucagon resistance (GR) through amino acid (AA) metabolism, reducing ureagenesis and resulting in hyperaminoacidemia. Increased AAs stimulate glucagon production to compensate for hepatic GR, and a vicious cycle occurs (the liver-pancreas axis). This hyperglucagonemia also leads to increased hepatic glucose release. A global IR state results in hyperinsulinemia, which may enhance sodium reabsorption and increase sympathetic nervous system activity, contributing to hypertension. Inflamed dysfunctional AT leads to increased insulin resistance, the release of proinflammatory adipokines, and decreased levels of the anti-inflammatory agent adiponectin. In the liver, triglycerides and toxic metabolites induce lipotoxicity, mitochondrial dysfunction and endoplasmic reticulum stress, leading to hepatocyte damage, apoptosis, and fibrosis. These dysfunctional hepatocytes synthesize and secrete dipeptidyl peptidase 4 (DPP4), which promotes inflammation in AT macrophages and increased IR. *AA* amino acids, *AT* adipose tissue, *DPP4* dipeptidyl peptidase 4, *FFA* free fatty acid, *GR* glucagon resistance, *HDL* high-density lipoprotein, *IR* insulin resistance, *LDL* low-density lipoprotein, *MASLD* metabolic dysfunction-associated steatotic liver disease, *SAT* subcutaneous adipose tissue, *SNS* sympathetic nervous system, *VAT* visceral adipose tissue, *VLDL* very low-density lipoprotein. Pointed arrows indicate stimulation or enhancement, while blunt ends indicate inhibition or repression. Dashed arrows indicate progressive reductions in a pathway. [2] Adapted from Godoy-Matos et al.

Metabolic dysfunction-associated steatotic liver disease (MASLD) is the most common liver disease in the world and affects 30% of the population [1]. It comprises a spectrum of liver manifestations associated with metabolic and cardiovascular disorders, such as obesity and/or unfavorable fat distribution, insulin resistance, arterial hypertension, dyslipidemia, and type 2 diabetes (T2D). MASLD is recognized as a hepatic manifestation of metabolic syndrome [2], and the current pathophysiology is shown in Fig. 1.

Diabetes is an important risk factor for MASLD [3]. The global prevalence of MASLD in people with T2D has increased by 23.2%, reaching 68.81% in 2016–2021 [4]. Additionally, T2D seems to accelerate the progression of liver disease in the MASLD spectrum [3].

Curiously, a prospective study of adults with type 1 diabetes (T1D) and T2D who had undergone liver biopsy reported that those with T1D had a risk of developing cirrhosis and portal hypertension that was similar to that observed in people with T2D after adjustment for potential confounding variables [5]. MASLD affects up to ∼30–40% of adult individuals with T1D [6], and it is associated with an increased risk of prevalent chronic kidney disease and retinopathy in this population [7].

### Definitions

MASLD is characterized by increased liver fat content (exceeding 5% of the parenchyma) [2, 8] and can be classified as steatosis (when there is only excess fat in the liver, with no more than minimal inflammation) or steatohepatitis (when there is lobular inflammation and hepatocyte ballooning, with or without fibrosis) [9].

People with steatohepatitis may develop various degrees of fibrosis, progressing to cirrhosis (5%), possibly accompanied by complications such as portal hypertension or hepatocellular carcinoma. Among those who develop cirrhosis, the lifetime risk of hepatocellular carcinoma is estimated to be between 5 and 30%, depending on demographic and clinical factors such as etiology and stage [10].

Physicians who treat patients with MASLD may acknowledge the strong coincidence with cardiometabolic disease, including atherosclerotic cardiovascular disease [11]. The association between MASLD and cardiovascular disease has been well established, as it is the major cause of morbidity and mortality in the population with MASLD. A meta-analysis [12] of 16 observational studies comprising data from 34,043 individuals diagnosed with MASLD by liver biopsy and imaging showed that people with MASLD had a greater risk of fatal and/or nonfatal cardiovascular events than did those without MASLD (odds ratio [OR] 1.64; 95% confidence interval [CI] 1.26–2.13). A higher risk of cardiovascular events was directly associated with MASLD severity (OR 2.58; 95% CI 1.78–3.75) and remained significant after full adjustment for other risk factors. Given the common drivers, potential causal factors, and the increased rate of cardiovascular events, comprehensive cardiometabolic risk management is warranted in patients with MASLD, preferably in a multidisciplinary approach [11].

### New nomenclature and classification

The classical terms “nonalcoholic fatty liver disease” (NAFLD) and “nonalcoholic steatohepatitis” (NASH) emerged in the 1980s to describe the liver histology of a series of patients with advanced steatohepatitis not associated with alcohol consumption. Since then, these acronyms have been extrapolated to describe the spectrum of the disease itself, which has led to some criticism [13]. First, they were informed more about what this clinical condition was not (alcoholic) than about its true nature (metabolic). Second, many patients with predominantly metabolic steatosis also ingest alcohol, often erratically.

In a move toward recognizing the true etiopathogenesis of hepatic steatosis, an international consensus of experts [8] proposed a new classification system for this disease. Furthermore, the consensus suggested that the term NAFLD be replaced by “metabolic (dysfunction)-associated fatty liver disease” (MAFLD). Following this line of thought, the Brazilian Diabetes Society (*Sociedade Brasileira de Diabetes [SBD]*) pioneered the term “metabolic fatty liver disease” (*doença hepática gordurosa metabólica [DHGM]*) in its 2021 Guideline [14], recognizing that, more than “associated with metabolic dysfunction”, steatotic liver disease is *per se* metabolic.

**Fig. 2:**
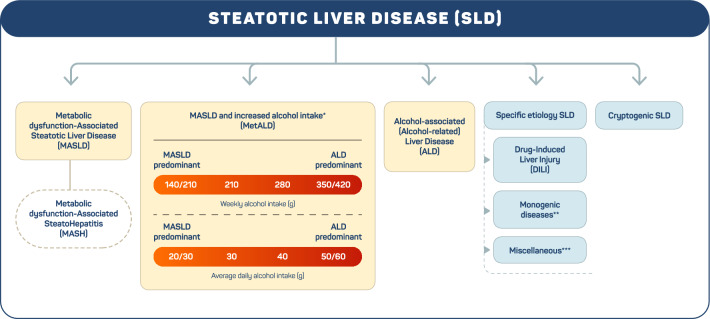
Steatotic liver disease (SLD) subclassification. This figure depicts the schema for SLD and its subcategories. Within the MetALD group, there was a continuum across which the contributions of MASLD and ALD varied. To align with the current literature, limits have been set accordingly for weekly and daily consumption, as the impact of varying levels of alcohol intake varies between individuals. *Weekly intake 140–350 g female, 210–420 g male (average daily intake of 20–50 g female, 30–60 g male). **e.g., lysosomal acid lipase deficiency (LALD), Wilson disease, hypobetalipoproteinaemia, inborn errors of metabolism. ***e.g., hepatitis C virus (HCV), malnutrition, celiac disease, human immunodeficiency virus (HIV). Adapted from Rinella et al. [15]

**Fig. 3:**
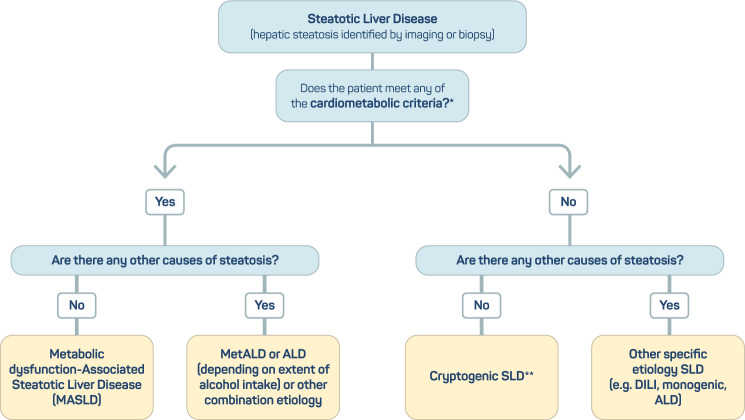
MASLD diagnostic criteria. In the presence of hepatic steatosis, the identification of any cardiometabolic risk factor can lead to a diagnosis of MASLD if there are no other causes of hepatic steatosis. If additional drivers of steatosis are identified, then this is consistent with a combination etiology. *At least 1 out of 5 cardiometabolic criteria: (1) body mass index ≥ 25 kg/m^2^ or waist circumference > 90 cm (M) and > 80 cm (F) or ethnicity adjusted equivalent; (2) prediabetes or type 2 diabetes; (3) blood pressure ≥ 130/85 mmHg or specific antihypertensive drug treatment; (4) plasma triglycerides ≥ 150 mg/dl or lipid lowering treatment; and (5) plasma HDL-cholesterol ≤ 40 mg/dl (M) and ≤ 50 mg/dl (F) or lipid lowering treatment. **In the absence of overt cardiometabolic criteria, other etiologies must be excluded, and if none is identified, this is termed cryptogenic SLD. *ALD* alcohol-associated/related liver disease, *DILI* drug-induced liver disease, *MASLD* metabolic dysfunction-associated steatotic liver disease, *MetALD* metabolic dysfunction and alcohol-associated steatotic liver disease, *SLD* steatotic liver disease.[15] Adapted from Rinella et al.

Although this change in classification and nomenclature was well accepted by the medical community in Brazil, internationally, the term “fatty” was still perceived pejoratively due to possible stigmatizing implications in the perception of patients and society in general about this condition. Furthermore, this change still did not allow for adequate subtyping of patients, which could pave the way for personalized medicine and better clinical trials. In 2023, a new consensus proposed by several international societies was published [15], establishing a new classification and nomenclature for fatty liver disease according to the schemes presented in Figs. 2 and 3.

In summary, the term “steatotic liver disease” (SLD) describes the most varied etiologies of fatty liver, and the term “steatohepatitis” was maintained. The presence of SLD associated with at least one of the five cardiometabolic risk factors defines MASLD, and the subgroup of patients who consumed significant amounts of alcohol is called MetALD. This subcategory allows a distinction to be made between individuals with pure MASLD and those with pure “alcohol-related liver disease” (ALD). The acronym “MASH” characterizes MASLD accompanied by steatohepatitis, and “cryptogenic SLD” defines SLD in people without cardiometabolic risk factors and without a known etiology for liver disease [15].

The new nomenclature is an opportunity to bring everyone together and spark new research to better understand epidemiology, natural history, diagnosis, biomarkers, and management strategies across the spectrum of SLD [16]. Notably, changing from NAFLD or MAFLD to MASLD led to a similar incidence of the respective steatotic liver disease. In the large cohort ELSA-Brasil, which included data from 10,651 individuals, the overall prevalence of NAFLD, MAFLD, and MASLD was 34.7% (95% CI 33.8–35.6, n = 3,697), 34.9% (95% CI 34.0–35.8, n = 3,718), and 33.4% (95% CI 32.6–34.4, n = 3,569), respectively [17].

By joining the national [18] and international communities in efforts to establish scientifically more appropriate and less stigmatizing diagnostic criteria and nomenclature and disseminate them globally, the SBD decided to adopt these new designations for the spectrum of steatotic liver disease in this guideline. Hereinafter, the new designations will be used to refer to all studies, including those that used the previous classifications and nomenclature.

## Methodology

This review is an English-translated update of part of the 2021 SBD Guidelines, and the methodology was approved for publication by the internal institutional steering committee. In brief, the SBD appointed the experts of the central committee, which regulated the methodology, reviewed the manuscripts, and judged the degree of recommendations and level of evidence. The SBD Metabolic Syndrome and Prediabetes Department drafted the manuscript, selecting key clinical questions for a narrative review using MEDLINE via PubMed and the MeSH terms [diabetes] and [fatty liver]. The best available evidence was reviewed, including randomized clinical trials (RCTs), meta-analyses, and high-quality observational studies related to MASLD diagnosis and treatment.

### Level of evidence

Three levels of evidence were considered: A—Data from more than one RCT or a meta-analysis of RCTs with low heterogeneity (I^2^ < 40%). B—Data from a meta-analysis with high levels of heterogeneity (I^2^ ≥ 40%), a single RCT, a prespecified subgroup analysis, large observational studies, or meta-analyses of observational studies. C—Data from small or nonrandomized studies, exploratory analyses, other guidelines, or expert consensuses.

### Degree of recommendation

For each defined recommendation, a poll was sent to all experts from the Metabolic Syndrome and Prediabetes Department and from the central committee. The frequency of the responses was analyzed, and a degree of recommendation was obtained based on the following criteria: I—More than 90% of the panel agreed; IIa—between 70 and 90% of the panel agreed; IIb—between 50–70% of the panel agreed; and III—Most of the panelists advised against a specific intervention. The terminology for the four degrees of recommendation was as follows: I—IS RECOMMENDED; IIa—SHOULD BE CONSIDERED; IIb—MAY BE CONSIDERED; and III—IS NOT RECOMMENDED.

### Screening and management

**Fig. 4:**
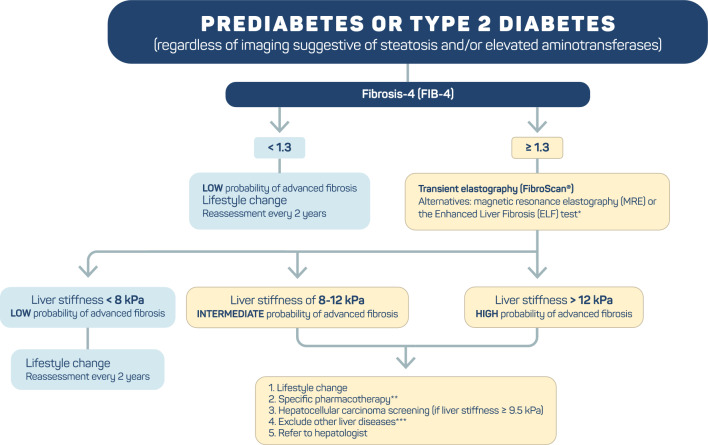
Screening and management of MASLD in patients with prediabetes or type 2 diabetes. *Depending on availability. **Drugs with proven benefits in MASLD. ***See Table 1

The strategy for screening and managing MASLD in people with T2D is depicted in Fig. 4 and Table 1, and it is addressed and detailed in the following recommendations. There is high heterogeneity in liver outcomes and measurements and outcomes, including histological (steatosis, steatohepatitis, and fibrosis), imaging/noninvasive, biochemical, and clinical liver parameters, among studies evaluating interventions for improving MASLD. In discussing the evidence that supports each of the following recommendations, the panel sought to emphasize studies with outcomes based on liver histology, whenever available, such as resolution of steatohepatitis without worsening of fibrosis, improvement of at least one fibrosis stage, and resolution of steatohepatitis with improvement of fibrosis.

**Table 1:** Laboratory tests for the differential diagnosis of MASLD

Anti-hepatitis C virus antibody Hepatitis B surface antigen Anti-hepatitis B surface antibody Anti-hepatitis B core antibody immunoglobulin G (IgG) Ferritin Transferrin saturation Ceruloplasmin Antinuclear antibody Antismooth muscle antibody Antiliver kidney microsome type 1 (anti-LKM1) Antimitochondrial antibody Alpha_1_-antitrypsin Immunoglobulin A (IgA) anti-tissue transglutaminase Serum IgA and IgG	

### Recommendations


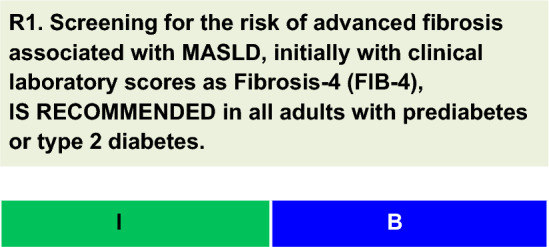

- Screening for the risk of advanced fibrosis is recommended in all adults with prediabetes or T2D, as MASLD is highly prevalent in this population. A meta-analysis with data from 80 studies involving 49,419 individuals revealed an overall MASLD incidence of 55.5% in patients with T2D [3]. According to a recent update of these data, the global MASLD incidence in patients with T2D has increased by 23.2% (p = 0.08), reaching 68.81% in 2016–2021 [4]. The pooled incidences of MASH and advanced fibrosis (≥ F3) were 66.44% and 15.49%, respectively [4].
- Another guideline also recommends screening for advanced fibrosis in high-risk populations, which includes people with prediabetes or T2D, since both conditions are important risk factors for poor prognosis in MASLD patients [19].
- Clinical laboratory scores, which include FIB-4 score, body mass index, aspartate aminotransferase (AST)/alanine aminotransferase (ALT) ratio, diabetes (BARD) score, aspartate aminotransferase-to-platelet ratio index (APRI), and NAFLD fibrosis score (NFS), are useful for risk stratification of advanced fibrosis (F3/F4 METAVIR) [20, 21]. Among these, the FIB-4 score has the best diagnostic accuracy [21]. It is available at the following link: https://www.hepatitisc.uw.edu/page/clinical-calculators/fib-4.
- The FIB-4 score was calculated from clinical and laboratory data, including age, ALT and AST levels, and platelet count [20]. At a cutoff value of 1.3, the FIB-4 score had a sensitivity of 84.4% and a specificity of 68.5% for detecting advanced fibrosis [21]. However, if FIB-4 is < 1.3, the risk of advanced fibrosis is ruled out, with a negative predictive value (the probability that a person with a negative test result is truly free of disease) of approximately 91% [21]. Patients classified as intermediate to high risk of advanced fibrosis should be followed up with other methods, as discussed below.

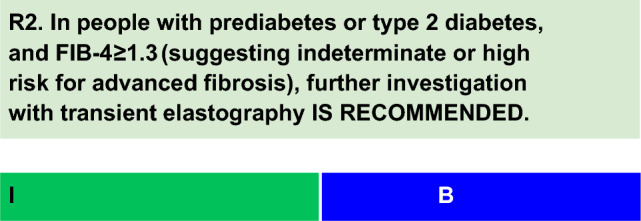

- Quantification of fibrosis and steatosis can be performed using liver elastography [22]. Elastography can distinguish the following stages of fibrosis: absent or mild (F0/F1), moderate (F2), advanced (F3), and cirrhosis (F4). Among ultrasound elastography methods, transient elastography (FibroScan^®^) is currently the most validated noninvasive technology [22].
- Sequential combinations of markers with a lower cutoff to rule out advanced fibrosis and a higher cutoff to rule out cirrhosis can reduce the false negative rate for advanced fibrosis. According to an individual patient data meta-analysis of 37 studies (n = 5,735; 33% with T2D), the sequential combination of FIB-4 (cutoffs: < 1.3; ≥ 2.67) and liver stiffness measurement by transient elastography (cutoffs: < 8.0 kPa; ≥ 10.0 kPa) had a sensitivity and specificity of 66% and 86%, respectively. This strategy resulted in a false negative rate of 9% for advanced fibrosis [23]. Upper cutoffs to rule-in cirrhosis (e.g., 12.0 kPa) could lead to a further decrease in the need for liver biopsies.
- Magnetic resonance elastography (MRE) has good accuracy in quantifying liver fat and assessing fibrosis. However, the high cost and low availability of these methods are limitations [24].
- The proper selection of methods for assessing fibrosis requires consideration of local availability. Noninvasive methods for examining MASH are not yet available.

**Important note: liver biopsy**

*Liver biopsy is the gold standard method for assessing steatosis, identifying MASH, and quantifying fibrosis. Because it is an invasive method that has its limitations in terms of cost, reproducibility, and risk of complications, it should be considered only in patients whose evaluation by noninvasive methods was doubtful, especially when the etiology of liver disease is unclear* [25].


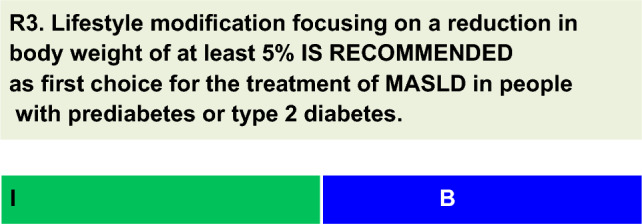

- Weight loss is the most effective measure for assessing histological improvement in MASLD patients. Losses of approximately 3% of body weight improve MASLD, but losses of more than 5% are necessary to reduce inflammation and stabilize fibrosis [26, 27].
- There was a dose‒response relationship between weight loss and the extent of histological improvement. A sustained weight loss of ≥ 7% can resolve MASLD in 65 to 90% of patients [20].
- Vilar-Gomez et al. [28] evaluated the liver histology of 293 subjects with MASLD treated with lifestyle modification (LSM). Losses of more than 10% of body weight promoted a reduction in the nonalcoholic fatty liver disease activity score (NAS) of 100%, resolution of MASLD of 90%, and regression of fibrosis of 45%. Notably, 66% of these individuals had alterations in glucose metabolism, and 33% were diagnosed with T2D [28].
- In the Look AHEAD study, which included people with T2D, an average weight reduction of 8% with LSM resulted in a significant reduction in liver fat, assessed by magnetic resonance, compared with that in the control group [29].
- Other studies in people with MASLD and T2D have shown similar results [30, 31].

**Important note: physical activity**

*Increased physical activity is associated with a reduction in all-cause mortality and cardiovascular mortality in individuals with MASLD* [32].


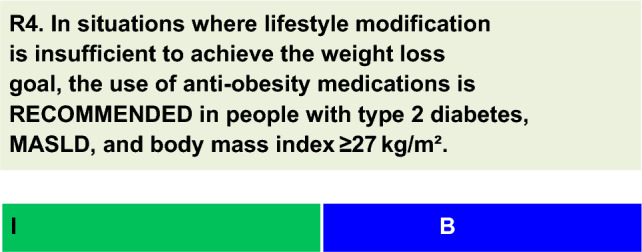

- In individuals with T2D, MASLD, or a body mass index (BMI) ≥ 27 kg/m^2^, when lifestyle measures are insufficient to promote the desired weight loss, additional pharmacotherapy for obesity is recommended [33].
- Some of the drugs approved for the treatment of obesity, i.e., orlistat [34], liraglutide 3.0 mg/day [35], and once-weekly semaglutide 2.4 mg [36, 37], have been investigated in patients with MASLD and T2D.
- In a 36-week RCT, adults with a BMI ≥ 27 kg/m^2^ and biopsy-proven MASH (n = 55; 7,2% with T2D) were randomized to receive a 1400 kcal/day diet plus vitamin E (800 IU/day) with or without orlistat (120 mg three times daily) [34]. Overall, orlistat did not enhance weight loss or improve liver enzymes, insulin resistance, or liver histopathology. Moreover, individuals who lost ≥ 5% of their body weight exhibited improved insulin resistance and steatosis, and those who lost ≥ 9% also achieved improved steatosis (p < 0.01), ballooning (p < 0.05), inflammation (p < 0.05), and NAS (p < 0.01) [34].
- Liraglutide 3.0 mg/day resulted in weight loss of 8.0 ± 6.7%, with 63% of patients achieving at least 5% weight loss [35, 38]. Liraglutide also ameliorates metabolic dysfunction, insulin resistance, and lipotoxicity, suggesting that some of the effects on MASLD may occur independently of weight loss [39].
- A systematic review and meta-analysis were conducted to evaluate the efficacy and safety of semaglutide in patients with MASLD; eight studies (data from 2413 participants) were included. Semaglutide at different doses (up to 2.4 mg once a week, the dose approved for the treatment of obesity) improved ALT (primary outcome) and reduced liver fat content (mean difference 4.97%; 95% CI 6.65 to − 3.29; p < 0.001) and liver stiffness (mean difference 0.96 kPa; 95% CI 1.87 to − 0.04; p = 0.04) [36]. Heterogeneity was moderate to high across all comparisons, and gastrointestinal symptoms and gallbladder-related diseases were high following semaglutide treatment compared to placebo (RR 1.54; 95% CI 1.02–2.34; p = 0.04) [36]. Importantly, this meta-analysis included the first MASH-related cirrhosis RCT evaluating once-weekly semaglutide 2.4 mg versus placebo [37]. In this study, the primary outcome of improvement in fibrosis without worsening of MASH was not reached, despite significant improvement in steatosis (measured by MRI), aminotransferases, weight, and hemoglobin A1c with semaglutide compared to placebo. No new safety concerns were raised [37].

**Important note: antidiabetic medications**

*In people with T2D and MASLD who have evidence of MASH and/or fibrosis, the use of pharmacotherapy specific to T2D acting on MASLD should be considered in conjunction with LSM for improvement of hepatic outcomes. Pioglitazone and the glucagon-like peptide-1 receptor agonists with proven benefit for MASLD are the first line therapies, according to the evidence described below and listed in Table* 2. *It is important to highlight that metformin is not associated with specific benefits in MASLD. In ten RCTs involving metformin use, only two small studies examined pre- and posttreatment outcomes. In both studies, despite improved glycemic control and a modest improvement in liver enzymes and ballooning compared with placebo, there was no benefit in improving liver stiffness, a surrogate for fibrosis* [40]. *Moreover, in a meta-analysis of five trials involving people with T2D and MASLD* [41], *metformin did not improve inflammatory, radiologic, and histologic parameters associated with MASLD, despite reductions in weight and hemoglobin A1c.*

**Table 2:** The main studies evaluating specific pharmacotherapy for T2D with benefit in MASLD

Intervention (daily dose)	Effect	Time	Refs.
Pioglitazone			
45 mg	LF reduction (58% vs*.* 17% in the PLB group); MASH resolution at 51%; improvement in the fibrosis score observed in LB	36 m	[44]
30–45 mg	Improvement of fibrosis (F3-F4 to F0-F2); MASH resolution in LB	6–24 m	[45]
30–45 mg	Reduction of disease activity score (NAS) in the evaluation by LB	every 6 m	[46]
GLP-1 receptor agonists (GLP-1RA)			
Liraglutide 1.8 mg	Resolution of MASH without worsening of fibrosis in LB	48 weeks	[48]
Semaglutide 0.4 mg	Resolution of MASH without worsening of fibrosis in LB	72 weeks	[51]
SGLT2 inhibitors^a^			
Empagliflozin 10 mg	MRI-assessed LF reduction (− 4.0% vs PLB), ALT improvement	20 weeks	[56]
Dapagliflozin 10 mg + omega 3	MRI-assessed LF reduction (− 21% vs PLB), improvement in liver biomarkers and enzymes (dapagliflozin monotherapy group)	12 weeks	[59]
Canagliflozin 100/300 mg	SH improvement compared to PLB or active comparator (meta-analysis with n = 6745); Smaller study with improvement of lobular inflammation, ballooning, and fibrosis (n = 9)	26 to 52 weeks	[60, 61]

*ALT* alanine aminotransferase, *GLP-1* glucagon-like peptide-1, *LB* liver biopsy, *LF* liver fat, *MASH* steatohepatitis, *MRI* magnetic resonance imaging, *NAS* nonalcoholic fatty liver disease activity score, *PLB* placebo, *SGLT2* sodium–glucose cotransporter-2

^a^To date, there are no studies evaluating histological liver outcomes associated with SGLT2 inhibitors

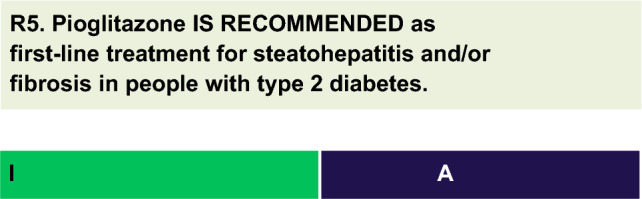

- Although there are no studies comparing antidiabetic medications in terms of MASLD-related outcomes such as cirrhosis and mortality, most studies comparing pioglitazone with placebo show improvements in inflammation and histologic changes [42].
- A systematic review and meta-analysis of six RCTs of people with T2D (n = 332) compared the effects of pioglitazone and other thiazolidinediones (TZDs) with those of a placebo or sulfonylureas in patients with MASLD. Compared with the placebo, the TZDs reduced liver fat by 6.6% (95% CI 12.56–0.96%; p = 0.022; I^2^ = 0%) [43].
- An RCT involving 101 people with prediabetes or T2D and biopsy-proven MASLD showed a 58% reduction in liver fat (p < 0.001) and improvements in ballooning, inflammation, and fibrosis scores (− 0.5; 95% CI 0.9–0.00; p = 0.039) with the use of 45 mg pioglitazone compared with placebo [44]. The study, which was originally designed for an 18-month follow-up period, showed that histologic and metabolic improvements persisted after 36 months of treatment. Adverse events did not differ between groups, except for weight gain (+ 2.5 kg) in the pioglitazone group.
- A meta-analysis of eight studies with TZDs involving individuals with and without T2D suggested that these agents may reduce advanced fibrosis (OR 3.15; 95% CI 1.25–7.93; p = 0.01; I^2^ = 0%) and resolve MASLD (OR 3.22; 95% CI 2.17–4.79; p < 0.001; I^2^ = 0%). The significance of this effect was limited to pioglitazone, and the results were similar when RCTs of people with T2D were excluded [45].
- A network meta-analysis compared the effects of different treatments on MASH; 48 RCTs and prospective studies were included. The primary endpoint was the reduction in the NAS associated with the use of different drugs with potential effects on MASLD. The most effective treatment for reducing the NAS per semester was pioglitazone (− 1.50; 95% CI − 2.08 to − 1.00). Pioglitazone is the best treatment for steatosis and reduces lobular inflammation [46].
- It is important to note that other factors should be considered when choosing to use pioglitazone for MASH patients with or without fibrosis, such as potential weight gain, the risk of bone fractures, and the presence of heart failure.

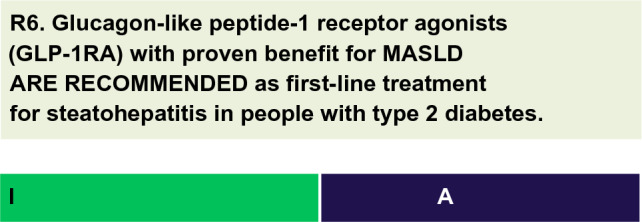

- To date, there is no evidence that GLP-1RA improve liver fibrosis. However, these agents promote histological benefits in MASLD, including reducing inflammation without worsening fibrosis [48, 49, 51, 52]. Furthermore, the role of GLP-1RA in the treatment of MASLD is not fully understood. Some studies have noted improvements in liver inflammatory markers and histologic changes associated with inflammation, but it is not possible to determine whether these effects are independent or secondary to weight loss.
- According to a meta-analysis of data from more than 4,000 people with T2D, liraglutide titrated to 1.8 mg/day significantly lowered liver enzymes in a dose-dependent manner compared to placebo [47].
- The LEAN study was a 48-week, multicenter, double-blind, placebo-controlled RCT in people with T2D and MASLD diagnosed by liver biopsy. The primary endpoint was MASLD resolution without worsening fibrosis. Nine (39%) of 23 people on liraglutide and two (9%) of 22 on placebo met the primary endpoint (risk ratio [RR] 4.3; 95% CI 1.0–17.7; p = 0.019). Two participants (9%) in the liraglutide group versus eight participants (36%) in the placebo group had fibrosis progression (RR 0.2; 95% CI 0.1–1.0; p = 0.04) [48].
- A systematic review of RCTs showed that GLP-1RA (liraglutide and exenatide) lowered liver enzymes and improved liver imaging and/or histology in patients with MASLD [49].
- A 104-week cardiovascular outcome study in people with T2D found significant reductions in ALT and ultrasensitive C-reactive protein with injectable semaglutide compared with placebo [50].
- A phase 2 RCT of semaglutide versus placebo lasting 72 weeks enrolled 320 patients with liver biopsy-proven MASH and fibrosis (F1 to F3). The primary outcome was the resolution of steatohepatitis without worsening fibrosis. Analysis of subjects with F2/F3 fibrosis showed that compared with placebo, subcutaneously administered semaglutide was significantly superior and resolved MASH in 40%, 36%, and 59% of subjects with daily doses of 0.1, 0.2, and 0.4 mg, respectively (OR 6.87; 95% CI 2.60–17.63; p < 0.001 for the 0.4 dose versus placebo). In this study, 69% of the participants had T2D, and the results in this subgroup were like those in the participants without T2D [51].
- A systematic review identified 11 RCTs that examined the use of GLP-1RA in patients with MASLD, totaling 936 individuals (70% with T2D). After 26 weeks of treatment, GLP-1RA promoted improvements in liver enzymes (especially ALT), a reduction in liver fat estimated by MRI (-3.92%; 95% CI -6.27 to -1.56), and improvements in inflammation without worsening fibrosis (OR 4.06; 95% CI 2.52–6.55; I^2^ = 0%, for semaglutide and liraglutide only) [52].
- A network meta-analysis examined the long-term effectiveness of daily and weekly formulations of GLP-1RA in people with MASLD and T2D [53]. Fourteen RCTs were analyzed, including data from 1666 participants. Primary outcomes were liver fat content, AST, and ALT levels; secondary outcomes included weight loss and gamma-glutamyl transferase (GGT) levels. The surface under the cumulative ranking curve (SUCRA) was used to rank the interventions. Twice daily exenatide ranked first (SUCRA 68%) for improvement in liver fat content, and weekly semaglutide ranked second (SUCRA 60%). Among the improvements in AST and ALT levels, once-daily semaglutide had the greatest improvement (SUCRA 100% and 96.5%, respectively); for weight loss, weekly semaglutide was the most effective (SUCRA 99.8%). In general, daily preparations seemed better for MASLD, and daily semaglutide may be the most effective treatment for MASLD and T2D compared with liraglutide, dulaglutide, exenatide, or placebo. Few studies evaluating weekly preparations were included, which may have limited the results [53].

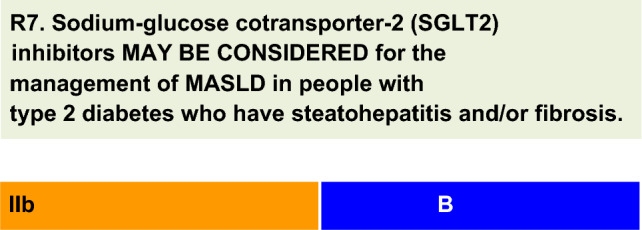

- Studies of the effects of SGLT2 inhibitors on MASLD outcomes are scarce. Small studies have shown reductions in liver enzymes and liver stiffness evaluated by elastography [54].
- A systematic review including data from four RCTs and four observational studies lasting at least 12 weeks evaluated the effect of SGLT2 inhibitors on liver enzymes in people with T2D and MASLD [55]. There was a significant decrease in ALT in seven studies, and most studies showed a decrease in AST and GGT levels. SGLT2 inhibitors were associated with a significant reduction in liver fat content, and of the three studies that assessed liver fibrosis indexes, two demonstrated significant improvement [55].
- In the E-LIFT study, fifty people with MASLD and T2D were randomly assigned to receive empagliflozin (10 mg/day) or maintain standard treatment for 20 weeks. Empagliflozin decreased liver fat content measured by MRI and improved ALT levels but not GGT or AST levels [56].
- Results from RCTs in patients with T2D showed a consistent reduction in aminotransferases with empagliflozin, in a pattern (reductions in ALT > AST) consistent with a reduction in liver fat content, regardless of changes in weight or hemoglobin A1c [57].
- An open-label RCT evaluated the use of dapagliflozin versus standard treatment for 24 weeks in people with T2D and MASLD. There were significant improvements in ALT, GGT, and liver stiffness measured by elastography in the dapagliflozin group. Dapagliflozin also reduced AST levels and attenuated fibrosis in a subgroup of patients with significant liver fibrosis (measured liver stiffness ≥ 8.0 kPa) [58].
- The EFFECT-II study investigated the effects of dapagliflozin (10 mg/day), omega-3, and a combination of both compared with placebo on liver fat content measured by MRI in subjects with T2D and MASLD over 12 weeks. All the active treatments significantly reduced the liver fat content from baseline, but only the combination treatment reduced the liver fat content (p = 0.046) and total liver fat volume (p = 0.037) compared with the placebo. Dapagliflozin monotherapy, but not combination therapy, decreased the levels of several biomarkers of liver injury, including ALT, AST, and GGT levels [59].
- A systematic review and meta-analysis of RCTs evaluated the effects of canagliflozin (100 mg/day or 300 mg/day) on liver enzymes in people with T2D. Eleven placebo-controlled or active-controlled studies were selected (n = 6,745). Canagliflozin significantly decreased the serum concentrations of ALT, AST, and GGT at 26 and 52 weeks, suggesting that it has a beneficial effect on the liver [60]. In addition, in a small prospective, uncontrolled study, liver biopsies were performed on nine patients with MASLD syndrome and T2D at baseline and after 24 weeks of canagliflozin treatment (100 mg/day). Histologic improvement occurred in all the patients. Steatosis, lobular inflammation, ballooning, and fibrosis scores decreased by 78, 33, 22, and 33%, respectively, at week 24 compared with baseline [61].

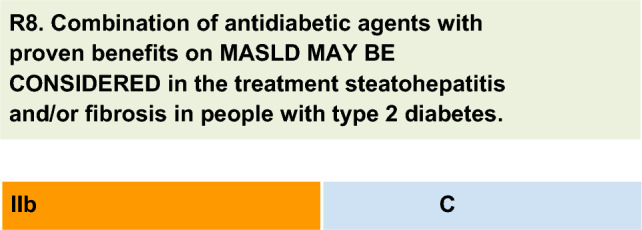

- This recommendation is based on expert opinion. Combined treatment of T2D is recommended to achieve satisfactory glycemic control. In this line, it seems reasonable to combine drugs that can additively or synergistically contribute to the resolution of liver disease for people with T2D who have MASH and/or fibrosis. Therefore, the panel suggests that combining pioglitazone with GLP-1RA (preferably) or one or both with SGLT2 inhibitors may be considered for people with T2D and MASLD who have evidence of MASH and/or fibrosis based on the potential benefits and lack of harm observed with combination therapy used for glycemic control and for improvement of non-histological liver outcomes [62]. The choice of drug combination should consider the benefits in diabetes control, weight management, and cardiovascular outcomes. Clinical trials comparing combination therapy with any of the drugs used as monotherapy for histological liver outcomes are ongoing [62].

**Important note: vitamin E, pentoxifylline, and silymarin**

*There is insufficient evidence to recommend the use of vitamin E, pentoxifylline, and silymarin for the treatment of MASLD in people with T2D.*

*In the PIVENS study* [63], *conducted in people with MASH and without T2D, vitamin E intake (800 IU/day) over a 2-year period resulted in an improvement in NAS score of two or more points, with no increase in fibrosis compared with placebo (43 vs. 19%, p < 0.001). Although vitamin E supplementation is being considered for MASH in people without T2D, specific studies in people with diabetes are still needed. A safety issue on vitamin E is that it seems to increase the risk of prostate cancer in elderly men* [64].

*In a small single-center, open-label RCT, including 35 patients with biopsy-proven MASH (28% with T2D), pentoxifylline 400 mg three times daily for 1 year determined improvement of NAS score compared to placebo (2.10 ± 1.07 vs. 0.90 ± 0.99, p < 0.001) *[65]. *Fibrosis, however, did not improve, and further data obtained from large series of patients are needed to assess pentoxifylline effectiveness.*

*In a double-blind, placebo-controlled RCT in patients with biopsy-proven MASH (n = 99; 53.5% with T2D), silymarin 700 mg three times daily for 48 weeks did not reduce the primary efficacy outcome, i.e., a decrease of 30% or more in NAS compared to placebo (32.7% vs 26.0%, p = 0.467) *[66].


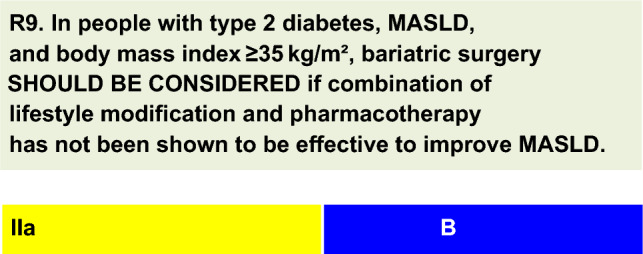

- In people with class III obesity, poorly controlled T2D, and poor response to LSM and drug therapy, bariatric surgery is an option for reducing weight and long-term metabolic complications [67, 68]. The benefits of bariatric surgery for MASLD have been consistent in several studies examining different surgical techniques, such as sleeve gastrectomy (SG), Roux-en-Y gastric bypass (RYGB), and adjustable gastric banding (AGB) [69–71].
- In a study of 1236 individuals with class III obesity, 32.6% had T2D, and MASLD improved with both RYGB and AGB. RYGB resulted, however, in greater weight loss (26% versus 21%) and provided more benefits for MASLD, glycemic parameters, and lipid metabolism, even though the baseline BMI was greater and MASLD was more severe in the RYGB group [69].
- A retrospective cohort study [72] investigated remission of MASLD after metabolic surgery, which included 252 patients with obesity, BMI ranging from 30 to 35 kg/m^2^, and at least one associated comorbidity, such as MASLD (n = 69) and/or T2D (n = 10). During the 3-year follow-up period, MASLD regressed in 84.6% of patients. T2D also regressed in 60% of patients, and disease control improved in 40%.
- Bariatric procedures provide long-term resolution of MASH and regression of fibrosis, as demonstrated in a 5-year prospective study including 180 patients with severe obesity and biopsy-proven MASH (71% with T2D) [73]. Liver biopsies were planned approximately 1 year and 5 years after bariatric surgery. At 5 years, MASH was resolved without worsening fibrosis in 84% of patients. Compared with those at baseline, 70.2% of the patients had fibrosis, which disappeared in 56% of the patients. Resolution of MASH was observed at 1 year after bariatric surgery in biopsies from 84% of patients, with no significant recurrence occurring between 1 and 5 years (p = 0.17). Fibrosis began to decrease by 1 year after surgery and continued to decrease until 5 years (p < 0.001). Notably, in this study, very few patients had cirrhosis [73].
- In a prospective study, 66 patients with advanced MASH (36 with advanced fibrosis and 30 with high activity grade without advanced fibrosis) underwent bariatric surgery and agreed to a follow-up liver biopsy at 6 ± 3 years. Bariatric surgery induced major histological improvement: 29% of patients had normal histology at follow-up biopsy, 74% had MASH resolution without fibrosis progression, and 70% had ≥ 1 stage of fibrosis regression. Despite MASH-related resolution, advanced fibrosis persisted in 47% of patients. These patients had lower weight loss and reduced hypertension or T2D remission rates [74].
- The BRAVES study was a multicenter, open-label RCT designed to compare bariatric metabolic surgery (RYGB or SG) versus lifestyle intervention plus medical care in patients with a BMI of 30–55 kg/m^2^ and biopsy-proven MASH (31.9% with T2D) [75]. According to the intention-to-treat analysis, MASH resolution without worsening of fibrosis (primary endpoint) was significantly greater in the RYGB and SG groups (56% and 57%, respectively) than in the LSM group (16%; p < 0.0001). Compared with that in the LSM group, the probability of MASH resolution was 3.60 times greater in the RYGB group and 3.67 times greater in the SG group. It is worth noting that, in the LSM group (n = 96), at baseline, there were only 34 patients under pioglitazone and 34 patients under liraglutide, and these numbers remained practically stable throughout the study [75].

**Important note: bariatric surgery and cirrhosis**

*People with T2D, MASLD, and cirrhosis should be carefully evaluated for indication for bariatric surgery based on case reports of decompensation of liver failure. A careful assessment of portal hypertension is indicated in patients with signs of cirrhosis before bariatric surgery* [76]. *Splenomegaly associated with thrombocytopenia and the presence of gastroesophageal varices on endoscopy can be considered surrogate signs of clinically significant portal hypertension and relative contraindications to surgery.*

## Conclusion

MASLD is the most common liver disease in the world and comprises a spectrum of liver manifestations associated with metabolic and cardiovascular disorders. Prediabetes and T2D are important risk factors for MASLD and accelerate its progression. Therefore, it is crucial to establish recommendations for screening and treatment of MASLD in people with prediabetes or T2D. Table 3 summarizes the final recommendations of the SBD for the management of MASLD in this special population.

**Table 3:**
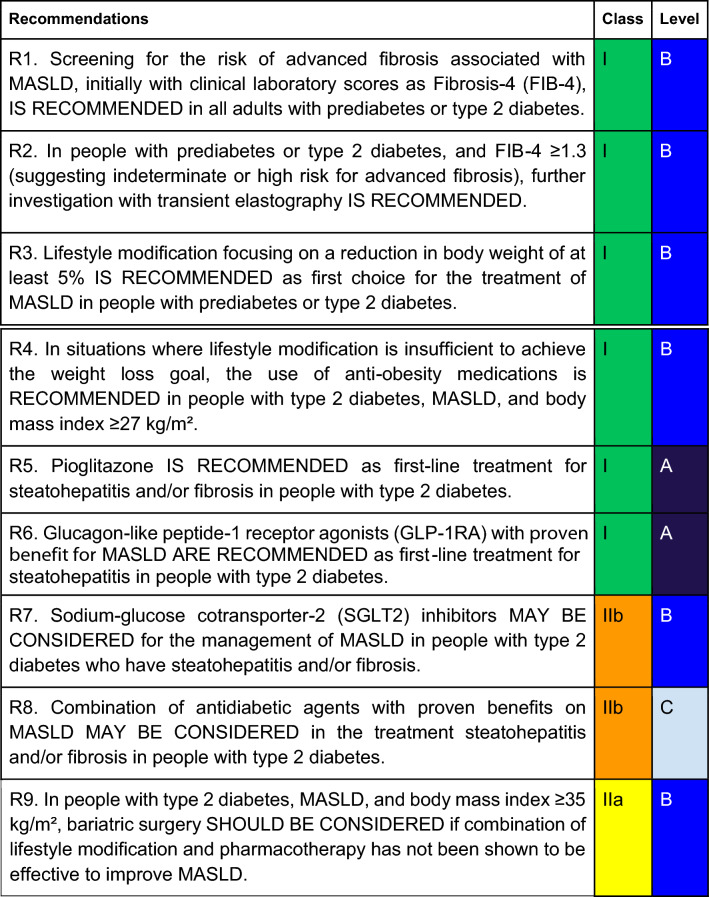
Summary of recommendations

## Data Availability

Not applicable.
