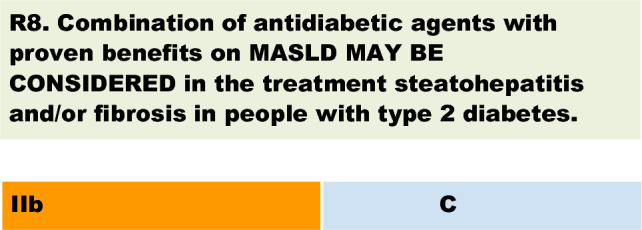# Correction: 2024 UPDATE: the Brazilian Diabetes Society position on the management of metabolic dysfunction‑associated steatotic liver disease (MASLD) in people with prediabetes or type 2 diabetes

**DOI:** 10.1186/s13098-024-01286-z

**Published:** 2024-02-28

**Authors:** Amélio F. Godoy‑Matos, Cynthia Melissa Valério, Wellington S. Silva Júnior, João Marcello de Araujo‑Neto, Marcello Casaccia Bertoluci

**Affiliations:** 1 Sociedade Brasileira de Diabetes (SBD), São Paulo, Brazil; 2 Instituto Estadual de Diabetes e Endocrinologia do Rio de Janeiro (IEDE), Rio de Janeiro, RJ Brazil; 3 Endocrinology Discipline, Department of Medicine I, Faculty of Medicine, Center of Biological Sciences, Universidade Federal do Maranhão (UFMA), Praca Goncalves Dias, 21, Centro, São Luís, MA CEP 65020‑240 Brazil; 4 Universidade Federal do Rio de Janeiro (UFRJ), Rio de Janeiro, RJ Brazil; 5 Universidade Federal do Rio Grande Do Sul (UFRGS), Porto Alegre, RS Brazil

**Correction: Diabetology & Metabolic Syndrome (2024) 16:23** 10.1186/s13098‑024‑01259‑2

The colour of the recommendation 8 should be changed from green to orange and the number to be changed from 1 to 11b. The original article [1] has been corrected.

Changed from 
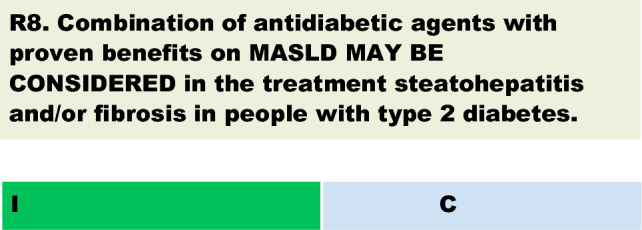
 to